# Comparative Study of Epidural Dexmedetomidine, Fentanyl, and Tramadol as Adjuvant to Levobupivacaine for Lower Limb Orthopedic Surgeries

**DOI:** 10.7759/cureus.25225

**Published:** 2022-05-22

**Authors:** Usha Shukla, Dheer Singh, Jheelam Singh, Jay Brijesh Singh Yadav

**Affiliations:** 1 Anaesthesiology and Critical Care, Uttar Pradesh University of Medical Sciences, Etawah, IND; 2 Anaesthesiology, Uttar Pradesh University of Medical Sciences, Etawah, IND

**Keywords:** tramadol, fentanyl, dexmedetomidine, levobupivacaine, epidural

## Abstract

Background

Dexmedetomidine, fentanyl, and tramadol as an adjuvant to local anesthetics improve postoperative analgesia when used in epidural anesthesia. We aimed to compare the efficacy of dexmedetomidine, fentanyl, and tramadol as an adjuvant to levobupivacaine in epidural anesthesia.

Materials and methods

This was a double-blinded randomized clinical trial (RCT). One-hundred twenty patients of either sex, aged 18-60 years, American Society of Anesthesiologists (ASA) physical status classification I and II, undergoing elective orthopedic procedures under epidural anesthesia were allocated into four groups of 30 each. The dexmedetomidine group received 15 ml of 0.5% levobupivacaine and 25 μg in 2 ml of dexmedetomidine, the fentanyl group received 15 ml of 0.5% levobupivacaine and 50 μg in 2 ml of fentanyl, the Tramadol group received 15 ml of 0.5% levobupivacaine and 100 mg of tramadol in 2 ml, and the control group received 15 ml of 0.5% levobupivacaine and 2 ml normal saline. Patients were monitored for the total duration of analgesia, time of first analgesic requirement, time to reach the T-10 level of sensory block, two-segment regression time of the sensory block, time to reach the motor block (Bromage 3), time to motor regression (Bromage 2), visual analog scale (VAS) scores at 0, 15 minutes, 30 minutes, and the first, second, sixth, twelfth, and twenty-fourth hours postoperatively, total analgesic consumption in 24 hours, and complications, if any, were recorded.

Results

During the inter-group comparison, VAS scores were lower, the duration of analgesia was longer, and the total analgesic consumption was less in the dexmedetomidine group compared to the fentanyl, tramadol, and control groups. The time to onset of sensory block to T-10 and the attainment of motor block up to Bromage 3 was lower in the dexmedetomidine group. Two segment regression and regression of motor block to Bromage score 2 was lowest for the dexmedetomidine group compared to the other groups. A lower incidence of hypotension and bradycardia was noted with dexmedetomidine.

Conclusions

Dexmedetomidine is the better alternative as an adjuvant to epidural anesthesia, with faster onset, good quality, and prolonged duration with no relevant adverse effects.

## Introduction

Regional anesthesia techniques are increasingly trending for upper and lower limb surgeries, as these provide optimal anesthetic care, which includes postoperative analgesia and improved patient satisfaction [1]. Epidural analgesia provides superior analgesia compared with other postoperative analgesic techniques such as general anesthesia. Additionally, perioperative epidural analgesia confers physiologic benefits, which may potentially decrease perioperative complications and improve postoperative outcomes. Levobupivacaine, an amide local anesthetic, is safe for regional anesthetic techniques because it has fewer hemodynamic changes and hastens the recovery and mobilization of the patients. Its low lipid solubility leads to greater sensory-motor differentiation by blocking sensory nerve fibers more rapidly than motor fibers [2-3].

Adjuvants like ketamine and fentanyl prolong the duration of sensory-motor block and decrease the cumulative dose requirement of local anesthetics. The co-administration of adjuvants has the potential to improve the efficacy of perineural blocks and decrease local anesthetic toxicity. Various opioids like fentanyl have been used as an adjuvant for epidural administration in combination with local anesthetic drugs to obtain a good anesthetic effect [4]. Fentanyl is a phenylpiperidine-derivative synthetic opioid agonist that is structurally related to meperidine. As an analgesic, fentanyl is 75 to 125 times more potent than morphine. Tramadol is a synthetic 4-phenyl-piperidine analog of codeine, a synthetic opioid, and a racemic mixture of two enantiomers, with synergistic anti-nociceptive interaction. Its analgesic activity is mediated through an agonist action at all types of opioid receptors with some µ receptor selectivity [5]. Tramadol has a minimal respiratory depressant effect. Dexmedetomidine, a highly selective α-2 adrenergic agonist acts on pre and post-synaptic sympathetic nerve terminals and the central nervous system to decrease the sympathetic outflow and norepinephrine release, causing analgesia and sympatholytic and sedative effects.

The synergistic effects between epidural local anesthetic and opioids are well-known but evidence regarding the combination of local anesthetic with dexmedetomidine through the epidural route is lacking in the literature. There is no such study that compared the dose equivalence of these drugs. Considering the benefits of levobupivacaine and various adjuvants, this randomized study was considered to evaluate the clinical effects of dexmedetomidine, fentanyl, and tramadol when used as an adjuvant to epidural levobupivacaine for lower limb orthopedic surgeries.

## Materials and methods

Study design

This prospective, randomized, double-blind clinical trial was conducted after obtaining approval from the Institutional Ethics Committee of Uttar Pradesh University of Medical Sciences, Saifai, Etawah (Ref.No.:1661/UPUMS/Dean(M)/Ethical/2020-21E.C 76/2019-20) with the informed consent of the patient. 

Inclusion and exclusion criteria

One-hundred twenty patients of either sex, aged 35-65 years, body mass index (BMI) 18-30 kg/m^2^, American Society of Anesthesiologists (ASA) physical status classification I and II, and posted for lower limb surgery were included in the study. Patients with a bleeding disorder, anticoagulants, neurological deficit, known allergy to local anesthetics drugs, local infection at the proposed site, chronic renal failure, chronic analgesic abuse, and pregnant patients were excluded from the study.

Sample Size Calculation

Sample size calculation was done assuming 80% power, 5% significance level with 95% confidence interval and assumed standard deviation being 2.5 as well as the absolute error being 1.00, the total sample size calculated out of being 120 patients divided equally into four groups of 30 each. Patients were randomly allocated using sequentially numbered cards in sealed opaque envelopes to one of the following groups:

Dexmedetomidine group (n=30): 15 ml of 0.5% levobupivacaine +25 microgram dexmedetomidine in 2 ml.

Fentanyl group (n=30): 15 ml of 0.5% levobupivacaine +50 microgram fentanyl in 2 ml

Tramadol group (n=30): 15 ml of 0.5% levobupivacaine +100 microgram tramadol in 2 ml

Control group (n=30): 15 ml of 0.5% levobupivacaine + 2 ml normal saline.

Study procedure

After informed written consent was taken, patients were kept fasting for six hours and received a tablet of alprazolam 0.5 mg at night before the surgery. Upon arrival in the operation room, ASA standard monitors, including non-invasive blood pressure and pulse oximeter were attached and baseline parameters were recorded. The heart rate and rhythm were monitored from a continuous visual display of an electrocardiogram. An intravenous line with an 18G canula was secured and all the patients were preloaded with 15 ml/kg lactated ringer solution. Under all aseptic precautions, the L4 L5 interspace was identified in the sitting position and 1.5 ml of 2% lignocaine was injected over the skin to raise a weal. A Tuohy needle (18 G) was inserted and guided slowly until the epidural space was reached, which was confirmed with the loss of resistance technique using 2 ml of normal saline. After confirmation of the epidural space, an epidural catheter was inserted in the cephalad direction and secured at the 9 cm mark. A test dose of 3 ml lignocaine 2% with adrenaline (5 microgram/ml) was administered to rule out the intrathecal and intravenous placement of the catheter. Patients were monitored for any adverse effects. After three minutes, the patients received the study solution of either group according to the randomization schedule by epidural catheter. Patients were monitored throughout the surgery, at the time of application of block, and thereafter up to 24 hours after the surgery.

The following parameters, viz heart rate, systolic blood pressure, diastolic blood pressure, and mean arterial blood pressure along with oxygen saturation were also recorded at the baseline (0 minutes), fifth minute, 15th minute, 20th minute, 25th minute, 30th minute, 40th minute, 50th minute, 60th minute, and 90th minute. In the postoperative period, vitals were recorded at 15 minutes, 30 minutes, the second hour, third hour, sixth hour, 12th hour, and 24th hour. The sensory block was assessed by the pinprick test using a blunt-end 26-gauge needle at each minute. Data regarding time to achieve the sensory block at the T10 level, two-segment regression, total duration of sensory block, time taken to achieve complete motor block, regression to Bromage 3, total duration of motor block, total duration of analgesia, and total consumption of rescue analgesic were recorded in 24 hours. Patient pain was evaluated by the visual analog scale (VAS), a scale of 0 to 10, where 0 is no pain and 10 is very severe pain. If the patient experienced the intensity of pain as VAS >3, the intramuscular injection diclofenac (1.5 mg/kg) was administered up to a maximum of 150 mg administered in 24 hours.

The Likert verbal scale was used to assess the patient’s satisfaction immediately after surgery and 12 hours after surgery (1=extremely dissatisfied, 2=dissatisfied, 3=some what dissatisfied, 4=undecided, 5=somewhat satisfied, 6=satisfied, 7=extremely satisfied). Adverse events, such as bradycardia, hypotension, desaturation, nausea, vomiting, etc., were recorded and analyzed.

Statistical analysis

All data were recorded, summarized, tabulated, and statistically analyzed using the Statistical Package of Social Sciences (SPSS) program (Version 20; IBM Corp., Armonk, NY). The statistical analysis of quantitative data (Mean ± SD) between the groups was done by the student’s t-test. The statistical analysis of qualitative data (N%) between the groups is done by using the one-way analysis of variance (ANOVA) unpaired t-test. A p-value <0.05 was considered statistically significant.

## Results

**Figure 1:**
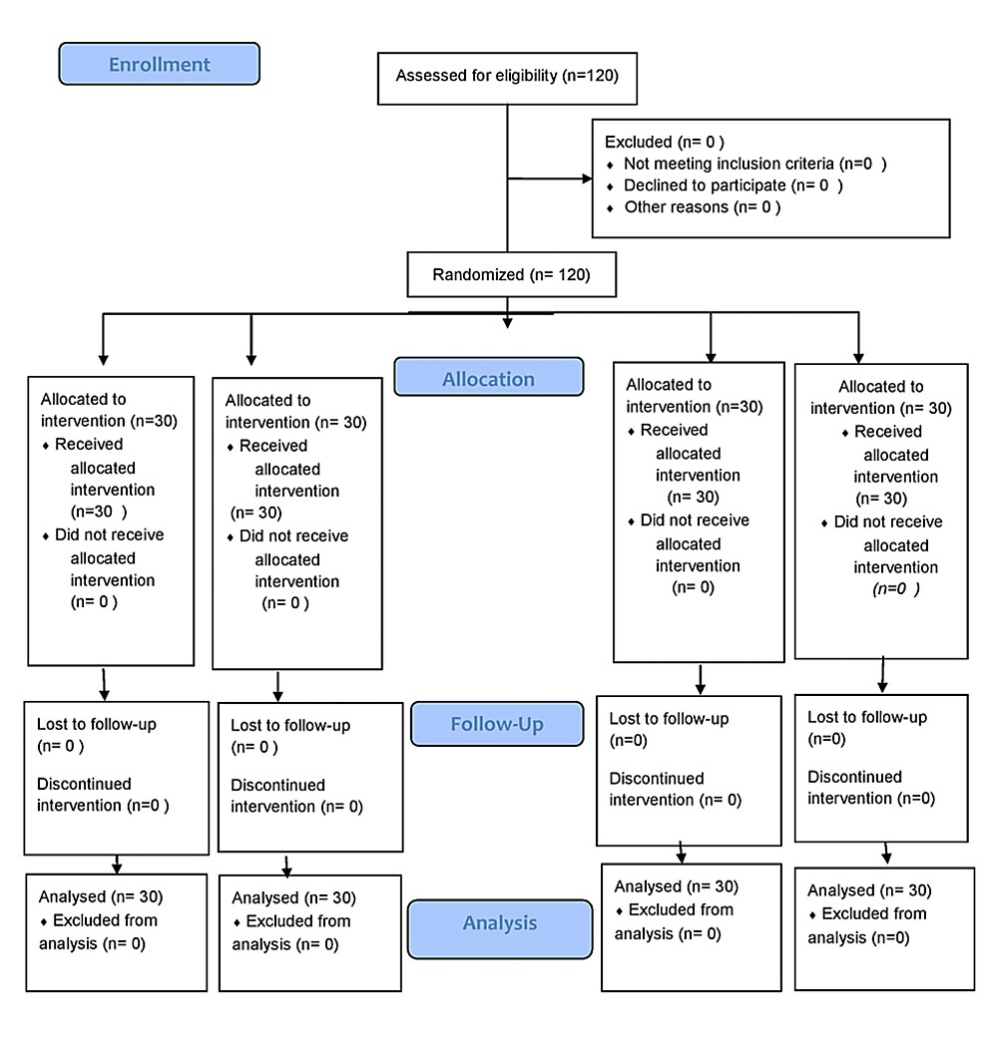
Consort flow diagram

None of the patients were excluded from our study (Figure 1). A total of 120 patients were enrolled in the study divided into four groups of 30 each. 

The demographic characteristics (age, weight, height, BMI, gender, ASA physical status classification) and duration of the study were comparable among the groups (Table 1).

**Table 1:** Demographic characteristics and duration of surgery in the studied groups (kg = kilograms, cms = centimeters, ! ANOVA test, * Student unpaired t-test; p > 0.05 = not significant; p < 0.05= significant; p < 0.001 = highly significant) ASA: American Society of Anesthesiologists; ANOVA: analysis of variance

Groups	Dexmedetomidine	Fentanyl	Tramadol	Control	p-value
	Mean ± SD	Mean ± SD	Mean ± SD	Mean ± SD	>0.05
^!^Age (years)	43.23 ± 15.64	39.57 ± 15.9	43.00±16.13	39.80 ±16.22	
*Gender (Male/Female)	20/10	21/9	23/7	23/7	
^!^Weight (kgs)	63.73 ±7.11	60.90 ±9.81	61.23 ±7.42	62.50 ±7.34	
^!^Height (cms)	167.07 ±7.81	165.17 ±6.29	165.97 ±6.47	164.83 ±6.72	
BMI (kg/m^2^)	22.83 ±1.94	22.32 ±3.27	22.25 ±2.62	23.02 ±2.5	
*ASA GRADE I/II	16/14	14/16	19/11	17/13	
^!^Duration of surgery (minutes)	69.33 ±14.19	71.00 ±15.72	69.33 ±13.44	66.00 ±13.8	

Time taken to reach the T10 level was shorter in the dexmedetomidine group (7.67±1.37 minutes), fentanyl group (9.53±1.7minutes), and tramadol group (11.13±2.21 minutes) and longest in the control group (19.57±4.49 minutes) (Table 2). During the inter-group comparison, the mean values were found to be statistically significant. (p<0.05). The two-segment regression time was slowest in the dexmedetomidine group (168.87±10.29 minutes), fentanyl group (135.63±8.92minutes), and tramadol group (131.13±7.28 minutes) and fastest in the control group (76.20±7.92 minutes). During the inter-group comparison, the mean values were found to be statistically significant (p<0.05). The total duration of sensory block was longest in the dexmedetomidine group (374.30±9.41 minutes), fentanyl group (255.70±11.03 minutes), tramadol group (171.52±35.26 minutes), and shortest in the control group (146.33±20.29 minutes). During the inter-group comparison, the mean values were found to be statistically significant. (p<0.05).

**Table 2:** Comparison of sensory block, time taken to reach the T10 level, two-segment regression, and total duration of sensory block among the groups (ANOVA test; p > 0.05 = not significant; p < 0.05= significant; p < 0.001 = highly significant. <0.01= highly significant) ANOVA: analysis of variance

Groups	Dexmedetomidine (D)	Fentanyl (F)	Tramadol (T)	Control (C)	p-value		
	Mean ±SD	Mean ±SD	Mean ±SD	Mean ±SD	D vs F	D vs T	F vs T
Time taken to reach the T10 level	7.67 ±1.37	9.53 ±1.7	11.13 ±2.21	19.57 ±4.49	<0.001	<0.001	0.001
2-segment regression time	168.87±10.29	135.63 ±8.92	131.13 ±7.28	76.20 ±7.92	<0.001	<0.001	0.018
Total duration (minutes)	374.30 ±9.41	255.70±11.03	171.52±35.26	146.33±20.29	<0.001	<0.001	<0.001

The time taken to reach the complete motor block was fastest in the dexmedetomidine group followed by the fentanyl group, tramadol group, and control group (21.63±2.34 minutes, 31.13±3.32 minutes, 50.80±5.16 minutes, and 59.87±3.25 minutes, respectively) (Table 3). During the inter-group comparison, the mean values were found to be statistically significant. (p<0.05). Regression to Bromage scale 1 was slowest in the dexmedetomidine group (179.90±10.35 minutes), fentanyl group (147.47±19.9 minutes), and tramadol group (111.60±5.22 minutes) and fastest in the control group (93.37±4.71 minutes). During intercomparison among the groups, the mean values were found to be statistically significant (p<0.05). The total duration of motor block was longest in the dexmedetomidine group (342.73±6.74 minutes), fentanyl group (239.87±7.55 minutes), and tramadol group (140.33±5.98 minutes) and shortest in the control group (125.80 ± 8.49 minutes). During intercomparison among the groups, the mean values were found to be statistically significant (p<0.05).

**Table 3:** Comparison of time taken to reach complete motor block, regression to Bromage scale 1, and total duration of motor block among the groups. (*student unpaired t-test; p > 0.05 = not significant; p < 0.05= significant; p < 0.001 = highly significant)

Groups	Dexmedetomidine (D)	Fentanyl (F)	Tramadol (T)	Control (C)	p-value		
	Mean ±SD	Mean ±SD	Mean ±SD	Mean ±SD	D vs F	D vs T	F vs T
Complete motor block (minutes)	21.63 ±2.34	31.13±3.32	50.80±5.16	59.87±3.25	<0.001	<0.001	<0.001
Regression to Bromage 1 (minutes)	179.90±10.35	147.47±19.9	111.60±5.22	93.37±4.71	<0.001	<0.001	<0.001
Total duration (minutes)	342.73 ±6.74	239.87±7.55	140.33±5.98	125.80±8.49	<0.001	<0.001	<0.001

The duration of analgesia is maximum in the dexmedetomidine group (462.20±9.72 minutes), fentanyl group (291.37±14.92 minutes), and tramadol group (211.17±17.3 minutes) and least in the control group (176.93±5.74 minutes) (Table 4). Intergroup comparison of the dexmedetomidine group with the fentanyl group and the tramadol group was found to be statistically significant. The demand for diclofenac as analgesia was the same in the tramadol group and the control group but the analgesic requirement was lower in the dexmedetomidine group.

**Table 4:** Comparison of duration of analgesia and analgesic consumption in 24 hours among the groups (* Student unpaired t-test; p > 0.05 = not significant; p < 0.05= significant; p < 0.001 = highly significant)

Groups	Dexmedetomidine (D)	Fentanyl (F)	Tramadol (T)	Control (C)	p-value			
	Mean ±SD	Mean ±SD	Mean ±SD	Mean ±SD	D vs F	D vs T	D vs C	F vs T
Duration of analgesia	462.20 ±9.72	291.37±14.92	211.17±17.3	176.93±5.74	<0.001	<0.001	<0.001	<0.001
Diclofenac (mg) in 24 hours	102.50±36.76	147.50±13.69	150.00 ±0	150.00±0	<0.001	<0.001	<0.001	0.161

Nausea was observed in the fentanyl group and in the tramadol group and was found not to be statistically significant. In the dexmedetomidine group, bradycardia was observed, was found to be statistically significant, and hypotension was also observed to be statistically insignificant. Table 5 shows the comparison of adverse effects among the groups.

**Table 5:** Comparison of adverse effects among the groups

Adverse Effects	Dexmedetomidine (n=30)	Fentanyl (n=30)	Tramadol (n=30)	Control (n=30)	p-value
	Percentage of patients	Percentage of patients	Percentage of patients	Percentage of patients	
Nausea	0.00	13.33	6.67	0.00	0.107
Vomiting	0.00	0.00	0.00	0.00	-
Bradycardia	16.67	0.00	0.00	0.00	0.001
Tachycardia	0.00	0.00	0.00	0.00	-
Hypotension	6.67	0.00	0.00	0.00	0.130
Hypertension	0.00	0.00	0.00	0.00	-

**Figure 2:**
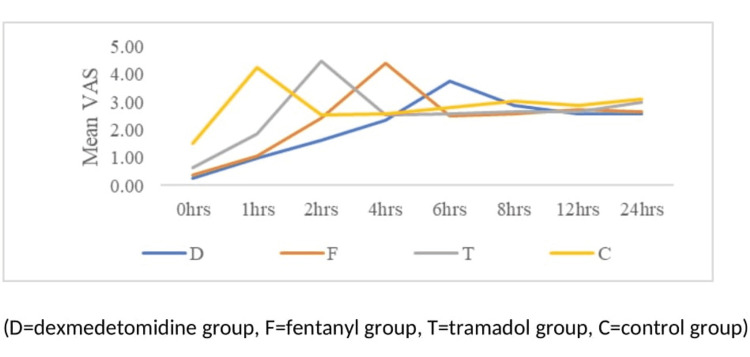
Comparison of visual analog scale among the groups

The baseline visual analog scale in the dexmedetomidine, fentanyl, tramadol, and control groups were 0.37±0.49, 0.37±0.49, 0.47±0.51, and 0.53±0.68, respectively, and were found to be comparable among groups. After one hour, the VAS score was higher in the control group (4.1±0.97); after two hours, VAS increased in the tramadol group (4.40±0.81) after four hours patients in the fentanyl group (VAS 4.4±0.5), and after six hours, VAS in the dexmedetomidine group was 3.77±0.9. Figure 2 shows a comparison of VAS among the groups.

**Figure 3:**
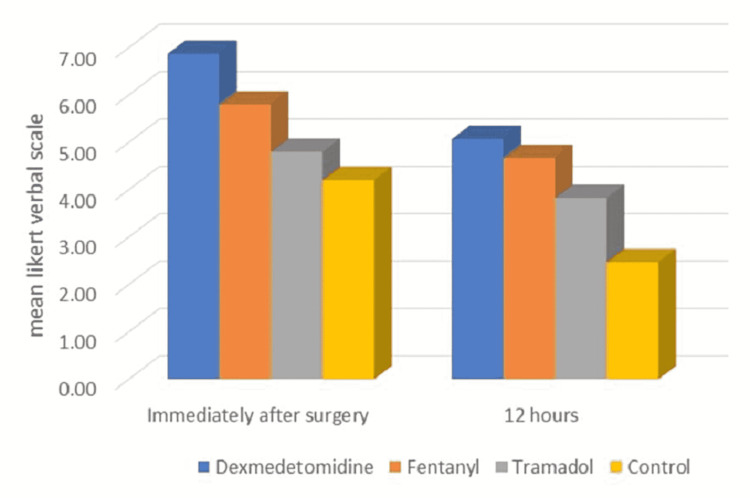
Comparison of Likert verbal scale among the groups

The patient satisfaction score immediately after the surgery in the dexmedetomidine group, fentanyl group, tramadol group, and control group were 6.87±0.35, 5.80±0.41, 4.80±0.41, 4.20±0.89, respectively. The patient satisfaction score 12 hours after surgery in the dexmedetomidine group, fentanyl group, tramadol group, and control group was 5.07±0.37, 4.67±0.61, 3.83±0.38, 2.47±0.51, respectively. There was a statistically significant difference in patient satisfaction scores among the groups (p<0.05). The patients’ satisfaction score in the dexmedetomidine group was better in comparison to the fentanyl group, tramadol group, and control group. Figure 3 shows the comparison of the Likert verbal scale among the groups.

## Discussion

In our study, the mean time of onset of the sensory blockade at the T10 level was fastest for dexmedetomidine followed by the fentanyl group and tramadol group, and slowest for the control group (p<0.001). This is consistent with the study done by Bajwa SJS et al. who observed early sensory blockade up to the T10 level in the dexmedetomidine group in a prospective, randomized, double-blind clinical study [6]. Similarly, Mahilamani PP et al. conducted a prospective, observational study and reported that the addition of dexmedetomidine to levobupivacaine leads to early sensory blockade at the T10 level (p=0.036) [7]. In our study, the mean time to two-segment regression of sensory blockade was the longest for the dexmedetomidine group compared to the remaining three groups. This is in line with the prospective, randomized study conducted by Paul A et al., who reported that the time for two-segment regression was longer with dexmedetomidine [8]. In concordance with our study, Sathyanarayana AL et al. observed that two-segment regression time was prolonged in the dexmedetomidine group as compared to the levobupivacaine group (p-value = 0.001) [9]. In the present study, the dexmedetomidine group had the longest sensory block duration followed by the fentanyl, tramadol, and control groups. Zainal R et al. [10] and Kaur S et al. [11] also reported that the total duration of sensory block duration was more in the dexmedetomidine group.

In our study, dexmedetomidine followed by the fentanyl, tramadol, and control groups took less time to achieve a complete motor block; this is in line with the prospective, randomized study conducted by Mahilamani PP et al., which reported that the group received levobupivacaine with dexmedetomidine took less time to achieve a complete motor block [7]. Similarly, Gupta K et al. found that the time taken to achieve a complete motor block was less in levobupivacaine with dexmedetomidine compared to levobupivacaine with the fentanyl group in a randomized prospective study for transurethral resection of the prostate [12]. In the present study, the mean time of regression of the motor blockade to Bromage scale 1 was late for the dexmedetomidine group. In line with our study, Bajwa SJ et al. observed that the time to regression to the Bromage scale 1 was more with the dexmedetomidine group compared to the clonidine group in a prospective randomized study [6]. Shaikh SI and co-workers also reported the meantime of regression of the motor blockade to the Bromage scale 1 was longer in the dexmedetomidine group as compared to the clonidine group [13]. In our study, the dexmedetomidine group had the longest motor block compared to the remaining three groups (p-value <0.001). Our study is also bolstered by many studies, namely Zainal R et al. [10], Zeng XZ et al. [14], and Gupta K et al. [12]. These studies showed the co-administration of dexmedetomidine and levobupivacaine results in prolonged motor block duration compared to levobupivacaine alone.

In the present study, the mean duration of analgesia was maximum for the dexmedetomidine followed by the fentanyl group, tramadol group, and control group. Pathak N et al., in a prospective, randomized, double-blind study, reported that the duration of analgesia was higher in patients administered levobupivacaine with dexmedetomidine as compared to levobupivacaine with fentanyl [15]. Similarly, Mansour FR et al. [16] and Saxena D et al. [17] found a longer duration of analgesia when tramadol with bupivacaine was used than with bupivacaine alone. In our study, overall, the VAS score was lower in the group that received dexmedetomidine as an adjuvant compared to the remaining groups. Our findings were supported by Paul A et al. in a prospective randomized study who reported a postoperative decrease in VAS score at 12, 18, and 24 hours in the dexmedetomidine control group as compared to the fentanyl group [8]. Similarly, Mohamed AA noted better pain relief (low VAS score) in patients of the dexmedetomidine group undergoing major abdominal cancer surgery [18]. The demand for diclofenac was the same in the fentanyl group, tramadol group, and control group but the analgesic requirement was lower in the dexmedetomidine group. In line with our study, Kejriwal A and coworkers compared the effect of clonidine and dexmedetomidine with levobupivacaine in the thoracic epidural block for laparoscopic cholecystectomy and reported that postoperative diclofenac requirement is less in the dexmedetomidine group [19].

In our study, a few complications were reported like nausea, itching, hypotension, and bradycardia. Nausea was observed in four patients in the fentanyl group, and two patients in the tramadol group. However, there was nothing statistically significant found among the groups. (p=0.107). In the dexmedetomidine group, hypotension was developed in two but it was not statistically significant. In the dexmedetomidine group, bradycardia was found in five patients and was found to be significant (p=0.001). Our observations were supported by Sathyanarayana AL et al., who conducted a randomized study and found bradycardia in the dexmedetomidine group (p=0.002) [9]. Also, Sinha S et al. studied the effect of dexmedetomidine on the paravertebral block using ropivacaine and reported episodes of bradycardia and hypotension with dexmedetomidine [20]. All the groups remained comparable in terms of hemodynamic parameters. One of the limitations of this study is that dexmedetomidine was not used according to the weight of patients. We did not measure the levels of dexmedetomidine and fentanyl in the plasma, which could have further supported the hypothesis that dexmedetomidine and fentanyl have a peripheral action rather than being centrally mediated.

## Conclusions

In this clinical trial, it can be concluded that the use of dexmedetomidine as an adjuvant to the local anesthetic agent during epidural block hastens the onset of sensory and motor blockade, provides a longer duration of analgesia, decreases the total analgesic requirement, and is not associated with nausea like fentanyl and tramadol, which cause clinically significant and unmanageable side effects.
